# Comparison of the PATHFAST D-dimer assay with two POC D-dimer assays

**DOI:** 10.1186/cc14401

**Published:** 2015-03-16

**Authors:** E Spanuth, B Ivandic, R Thomae, E Giannitsis

**Affiliations:** 1 DIAneering GmbH, Heidelberg, Germany; 2 Mitsubishi Chemical Europe, Düsseldorf, Germany; 3 University of Heidelberg, Germany

## Introduction

The early exclusion of PE is a major precondition for goal-oriented diagnostic and therapeutic measures. The aim of the study was to evaluate the new point-of-care assay PATHFAST D-dimer in comparison with VIDAS D-Dimer Exclusion and STRATUS CS D-dimer.

## Methods

A total of 272 patients with symptoms of PE and VTE were included. The diagnoses of VTE and PE were established by duplex ultrasound, venography and spiral CT. D-dimer values were determined in the patients and in plasma samples obtained from 102 healthy individuals who served as the control group.

## Results

Mean D-dimer concentration of the control group and of the patient group with PE was 0.28 (95% CI: 0.25 to 0.31) mg/l and 1.45 (95% CI: 1.23 to 1.72) mg/l, respectively. Receiver operator characteristics analysis revealed an optimized cutoff value of 0.466 mg/l for the PATHFAST D-dimer assay (AUC = 0.975 (95% CI: 0.938 to 0.993); sensitivity: 95% (95% CI: 86 to 99%); specificity: 89% (95% CI: 82 to 95%)). Therefore we used a rounded up cutoff value of 0.5 mg/l to examine the diagnostic accuracy of PATHFAST D-dimer to exclude PE. The correlation between PATHFAST and VIDAS Results was particularly close for concentrations at or around the critical cutoff value of 0.5 mg/l. The correlation between PATHFAST and STRATUS results was particularly close in the patient group with VTE (*r *= 0.9694), whereas slightly lower results were obtained with STRATUS in the control group. With the widely used cutoff value 0.5 mg/l, PATHFAST demonstrated suitable sensitivity but not STRATUS. ROC analysis indicated that optimal cutoff values could be set at either 0.5 or 0.6 mg/l and at 0.3 or 0.4 mg/l for PATHFAST and STRATUS, respectively.

## Conclusion

By use of the PATHFAST D-dimer assay only six of diagnoses were missed at the time of first presentation compared with 10 diagnoses missed by the VIDAS D-dimer Exclusion assay, yielding higher sensitivity of the PATHFAST D-dimer assay compared with the VIDAS assay (90% vs. 83%). The STRATUS assays showed comparable performance and appeared to be suitable for the exclusion of VTE in the emergency room setting, whereas PATHFAST demonstrated superior sensitivity. Moreover, the PATHFAST analyzer allows simultaneous determination of D-dimer and cardiac troponin I within 16 minutes from whole blood samples. Therefore, this method might be useful at the point of care for early diagnostic assessment of patients with symptoms of PE or chest pain admitted to the ER or to the chest pain unit.

